# Dominant Functional Group Effects on the Invasion Resistance at Different Resource Levels

**DOI:** 10.1371/journal.pone.0077220

**Published:** 2013-10-22

**Authors:** Jiang Wang, Yuan Ge, Chong B. Zhang, Yi Bai, Zhao K. Du

**Affiliations:** 1 School of Life Science, Taizhou University, Linhai, China; 2 Bren School of Environmental Science and Management/Earth Research Institute, University of California Santa Barbara, Santa Barbara, California, United States of America; University of Waikato (National Institute of Water and Atmospheric Research), New Zealand

## Abstract

**Background:**

Functional group composition may affect invasion in two ways the effect of abundance, i.e. dominance of functional group; and the effect of traits, i.e. identity of functional groups. However, few studies have focused on the role of abundance of functional group on invasion resistance. Moreover, how resource availability influences the role of the dominant functional group in invasion resistance is even less understood.

**Methodology/Principal Findings:**

In this experiment, we established experimental pots using four different functional groups (annual grass, perennial grass, deciduous shrub or arbor and evergreen shrub or arbor), and the dominant functional group was manipulated. These experimental pots were respectively constructed at different soil nitrogen levels (control and fertilized). After one year of growth, we added seeds of 20 different species (five species per functional group) to the experimental pots. Fertilization significantly increased the overall invasion success, while dominant functional group had little effect on overall invasion success. When invaders were grouped into functional groups, invaders generally had lower success in pots dominated by the same functional group in the control pots. However, individual invaders of the same functional group exhibited different invasion patterns. Fertilization generally increased success of invaders in pots dominated by the same than by another functional group. However, fertilization led to great differences for individual invaders.

**Conclusions/Significance:**

The results showed that the dominant functional group, independent of functional group identity, had a significant effect on the composition of invaders. We suggest that the limiting similarity hypothesis may be applicable at the functional group level, and limiting similarity may have a limited role for individual invaders as shown by the inconsistent effects of dominant functional group and fertilization.

## Introduction

Determining the factors that control the invasion of exotic species is important in invasion biology [1], [2]. Recent studies suggest that invasibility of plant communities can be influenced by a number of factors, including traits of invaders [1], [3]–[5], traits of dominant species [6]–[8] and resource availability [9], [10]. However, few studies have considered the interactions of these factors in determining effects on invasion [11], [12].

Functional group composition of the resident community has been proved critical to community resistance to invasion [13]–[16]. The limiting similarity hypothesis predicts that resident species most functionally similar to the invader should provide greater invasion resistance because of a greater overlap in resource use [14], [17]. Consequently, functional group composition should be a good predictor of invasion resistance [18]. However, prior studies have either supported [19]–[23] or contradicted [8], [24], [25] the limiting similarity hypothesis.

Functional group composition most likely affects invasion in two major ways: the effect of abundance, i.e. dominance of functional group; and the effect of traits, i.e. identity of functional groups [26]. Many previous studies focused on the effect of functional group identity on invasion [8], [14], [18], [27]. However, dominant functional groups in communities are known to strongly regulate community structure and ecosystem function [13], [15], [16]. Consequently, it is most likely that relative abundance, rather than presence, of certain functional groups may be a more powerful predictor of invasibility [8], [14]. At present, the relative roles of dominance and identity of functional group in invasion resistance remain little understood [26].

Evidence in support of limiting similarity is mainly from artificially established not natural communities [28]. Abiotic factors, such as variations in nutrient availability, disturbance and propagule pressure, which were controlled in these artificially established communities, were postulated as the reason for the mixed results [28]. However, how abiotic factors influence the role of limiting similarity remains little known. Davis and Pelsor [29] found increased resource availability tended to reduce competition intensity between resident species and invaders. Consistent with their result, many prior studies showed that high nutrient availability promoted community invasibility [29]–[31]. Consequently, we postulate that competitive exclusion of invaders by functionally similar resident species may be affected by resource availability.

Life form is the long-term adaptation performance of plants to environment, and plants of the same life form should have similar ways and methods of adapting to the environment [32]. In eastern China, many early successional communities established from disturbed plant communities and abandoned agricultural fields are hotspots of invasions in the area. Plants of these communities mainly belong to four life forms: annual grass, perennial grass, deciduous shrub or arbor and evergreen shrub or arbor. The resource use efficiency of these life forms differ significantly [33]. Moreover, the amounts of soil nitrogen in eastern China are consistently increasing due to atmospheric nitrogen deposition [34]. Here we consider how invasibility is influenced by the interactive effects of dominant functional group and fertilization (nitrogen addition). We created experimental communities that differed in dominant functional groups with two soil nitrogen levels, and controlled for functional group richness (four functional groups) and functional group identity. We used a total of 20 invader species: five species from each of four different functional groups (annual grass, perennial grass, deciduous shrub or arbor and evergreen shrub or arbor). We tested whether invading species had lower success in pots dominated by a functionally similar group, independent of functional group identity. We also tested whether the effects of the dominant functional group on invaders changed with resource availability and what mechanisms led to such change.

## Materials and Methods

### Ethics Statement

No specific permissions were required for this research. The pot experiment was conducted in the campus of Taizhou University, where some of the authors are staff members. The site of Taizhou University used for conducting the study is the experimental center for the authors. Consequently, use of this site did not require permission. The exotic species used in this study were either natives or common exotics with established local populations in the mountain area around Linhai city where Taizhou University is located. Consequently, all plant species used in this study are not endangered or protected.

### Experiment Design

We conducted an outdoor pot experiment at the campus of Taizhou University (28°3512N, 120°4791E) from June 2008 to May 2011. The region has a subtropical climate with an annual rainfall of 1800 mm, occurring mostly during spring and summer. The annual average temperature is 19.5°C. Photosynthetically active radiation (PAR, 9∶00–15∶00) was in the range of 600–2200 µmol m^−2^ s^−1^ at the experimental site. We established experimental pots using four different functional groups, each containing two native species that have high abundance in the mountain area around Linhai city of eastern China. These were (i) annual grass (AG): *Perilla frutescens* Linn., Britt and *Mazus pumilus* (Burm. f.) Van Steenis; (ii) perennial grass (PG): *Inula japonica* Thunb and *Plantago asiatica* Linn; (iii) deciduous shrub or arbor (D): *Grewia biloba* G. Don var. *parviflora* (Bunge) H.-M. and *Koelreuteria integrifoliola* Merr; and (iv) evergreen shrub or arbor (E): *Cinnamomum camphora* (L.) Presl and *Urena procumbens* Linn. The seeds of eight species were collected from the mountain area around Linhai city and were sown in trays in May 2008, and the seedlings were transplanted into plastic containers (pots, 80 cm×80 cm×60 cm) in June 2008, which were placed outdoors. Functional group richness and functional group identity of all pots were controlled, and only the dominant functional group was differently manipulated by varying the number of transplanted seedlings of different functional groups (Table 1). Each pot consisted of 32 plants. The plant density was similar to the natural density of plant communities in the mountain area around Linhai city. Plants of the same species were not transplanted adjacently, and 32 plants were evenly distributed in the pot. Six replicate pots were used for each dominant functional group treatment.

**Table 1:** The number of transplanted seedlings in different dominant functional group treatments.

	Dominant functional group treatment			
Resident species	AG	PG	D	E
AG				
* Perilla frutescens* (Linn.)	10	2	2	2
Britt and *Mazus pumilus* (Burm.f.) Van Steenis	10	2	2	2
PG				
* Inula japonica* Thunb	2	10	2	2
* Plantago asiatica* Linn	2	10	2	2
D				
* Grewia biloba* G. Don var. *parviflora* (Bunge) H.-M	2	2	10	2
* Koelreuteria integrifoliola* Merr	2	2	10	2
E				
* Cinnamomum camphora* (L.) Presl	2	2	2	10
* Urena procumbens* Linn	2	2	2	10

Footnotes: AG: annual grass; PG: perennial grass; D: deciduous shrub or arbor; E: evergreen shrub or arbor.

A total of 48 pots were established: 24 pots (four dominant functional group treatments × six replicates) were fertilized and the other 24 pots were not fertilized (i.e. control pots). The mountain yellow soil (all g kg^–1^: organic matter 1.64±0.17, total P 0.12±0.02 and total N 0.73±0.16) were used as substrate. The control pots were filled with a mixture of nutrient-poor sand and mountain yellow soil (sand:soil = 5∶1; the characters of mixture, all g kg^–1^: organic matter 0.68±0.12, total P 0.07±0.03 and total N 0.34±0.11). The concentration of total N was only about half of the natural soil N around Linhai city. The fertilized pots additionally received 6 g m^–2^ y^–1^ of N applied as ammonium nitrate at 2 g m^–2^ each time in July 2009, October 2009, March 2010, July 2010, October 2010 and March 2011. Except for two dead seedlings of *Cinnamomum camphora* (L.), all seedlings grew well before intentionally introducing the seeds of invader species. To maintain the composition of communities before seed addition of invader species, we hand-weeded each pot monthly in the first year. To simulate the assembly of natural communities, the seedlings of resident species were not removed after the seed addition of invader species. In addition to natural rainfall, the pots were uniformly watered during drier periods.

Twenty species were selected as invader species (Table 2) that were either natives from the plant communities in the mountain area around Linhai city or common exotics that had established local populations there; however, none were included in the original experimental pots. The seeds of these species were also collected in the mountain area of Linhai city in 2008. Hence, invasions were not limited by the invaders’ inability to survive the climate. These invader species were intentionally introduced into pots through the artificial ‘seed rain’ in June 2009. A total of 600 seeds, 30 seeds of each invader species, were mixed with 200 g soil, and then evenly sown in each pot. After seed additions, except for the resident and invader species, the seedlings of other spontaneously colonizing species were removed monthly. However, there were still seedlings of other species that spontaneously colonized the control and fertilized pots when the success of invasion was measured, which may be due to germination between hand-weeding operations (Table S1 in File S1). However, the coverage of these species only represented a very small proportion of total coverage of invader species (10.4 and 6.2% in the control and fertilized pots of 2010, respectively, and correspondingly 11.8 and 8.9% in 2011), and were not included in the data analysis. In May of 2010 and 2011, we measured the coverage and seedling numbers of invader species in each pot. To measure coverage, we overlaid a 100-cell grid (1 cm^2^ per cell in a 10×10 grid) over each pot and tallied the number of grid cells occupied by invader species.

**Table 2:** The sown invader species in the experimental pots.

Life form	Invader species	Abbreviation		Family	
Annual grass (AG)					
	*Celosia argentea* Linn	CA		Amaranthaceae	
	*Corchoropsis tomentosa*(Thunb.) Makino	CT		Tiliaceae	
	*Cerastium glomeratum* Thuill	CG		Caryophyllaceae	
	*Bidens bipinnata* Linn	BB		Compositae	
	*Triumfetta annua* Linn	TA		Tiliaceae	
Perennial grass (PG)					
	*Tubocapsicum anomalum*(Franch. et Sav.) Makino	TAM		Solanaceae	
	*Oxalis corniculata* Linn	OC		Oxalidaceae	
	*Reynoutria japonica* Houtt	RJ		Polygonaceae	
	*Polygonum perfoliatum* Linn	PP		Polygonaceae	
	*Gahnia tristis* Nees	GT		Cyperaceae	
Deciduous shrub or arbor (D)					
	*Rhus chinensis* Mill	RC		Anacardiaceae	
	*Vitex negundo* var. cannabifolia(Sieb.et Zucc.) Hand.-Mazz	VN		Verbenaceae	
	*Litsea cubeba* (Lour.) Pers	LC		Lauraceae	
	*Hibiscus mutabilis* Linn	HM		Malvaceae	
	*Euscaphis japonica* (Thunb.) Kanitz	EJ		Staphyleaceae	
Evergreen shrub or arbor (E)					
	*Pittosporum tobira* (Thunb.) Ait		PT	Pittosporaceae	
	*Syzygium buxifolium* Hook. et Arn		SB	Myrtaceae	
	*Photinia serrulata* Lindl. var. Serrulata		PS	Rosaceae	
	*Elaeagnus obovata* Li		EO	Elaeagnaceae	
	*Nandina domestica* Thunb		ND	Berberidaceae	

A PAR ceptometer (GLZ-C, Zhejiang Top Instrument Co., Ltd) was used to measure light availability in the control and fertilized pots. Two months after the seed addition of invader species, four points of each pot were randomly determined to measure the light transmittance percentage every month from August 2009 to May 2011 at midday on cloud-free days (Figure S1 in File S1). The PAR above the community canopy and the PAR at ground level were measured at the same point. The average light transmittance percentage (light transmittance percentage = 100×the PAR at ground/the PAR above community canopy) of four points was used to indicate light availability of each pot at each measured time. The average values from August 2009 to May 2010 were used to indicate light availability of each pot in 2010, and the average values from June 2010 to May 2011 in 2011.

### Statistical Analyses

We used indicator species analysis [8], [35], [36] to determine which invader species had the highest coverage and seedling numbers in pots dominated by which functional group. Indicator species analysis involved calculating a metric (indicator value, *IV*) that summarized both the relative abundance and the frequency of each invader species in each dominant functional group treatment.

First, the mean abundance of invader species in pots dominated by a certain functional group was calculated:
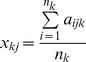
where 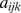 = abundance of invader species *j* in pot *i* dominated by functional group *k*, and 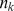 = number of pots dominated by functional group *k.*

Then the relative abundance 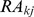 of invader species *j* in pots dominated by functional group *k* was calculated:
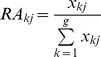
where *g* = total number functional groups.

Then relative frequency 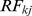 of invader species *j* in pots dominated by functional group *k* was calculated:
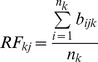
where 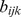 = the presence (1) or absence (0) of invader species *j* in pot *i* dominated by functional group *k.*

Then *IV* was calculated:
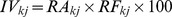



*IV* ranges from 0 (no presence of an invader species in a given dominant functional group treatment) to 100 (perfect indication). A perfect indication score (100) means that an invader species occurred only in a given dominant functional group treatment, and always in that treatment. Data of control and unfertilized pots were respectively analyzed. The observed *IV* was compared with an expected *IV* calculated using Monte Carlo randomizations of the data, where species frequency and abundance data from each pot were randomly assigned to a group/treatment 1000 times. The null hypothesis was that the observed *IV* was not larger than would be expected by chance (as calculated by the randomization procedure). The indicator species analyses were performed in PC-ORD 4.25 [37]. The *IV* of each invader species were analyzed by one-way ANOVA to determine whether the invader species had the lowest invasion success in pots dominated by the same functional group.

The effect of fertilization on the invader species was explored by calculating the relative effect index (REI):
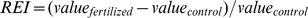
where value_fertilized_ is the coverage (seedling number) of invader species in the corresponding fertilized pots and value_control_ is the coverage (seedling number) of invader species in the corresponding control pots. All values for the control pots were plused 1 in order to guarantee computation.

Effects of fertilization and dominant functional group on the total coverage and seedling number of invaders, and on the coverage and seedling number of individual invaders, were evaluated using two-way ANOVA for years 2010 and 2011 separately. Effects of fertilization and dominant functional group on the coverage and seedling number of invaders of the same functional group were also evaluated using two-way ANOVA for 2010 and 2011 separately, and LSD tests detected differences in dominant functional group treatment means. The differences of *IV* and REI of invader species between pots dominated by the same functional group and pots dominated by another functional group were evaluated using independent-sample *t-*tests for the control and fertilized pots separately. We used ANCOVA to determine how dominant functional group, light availability, other invaders of the same functional group and invaders of other functional group influenced the coverage and seedling number of individual invader species.

## Results

**Figure 1:**
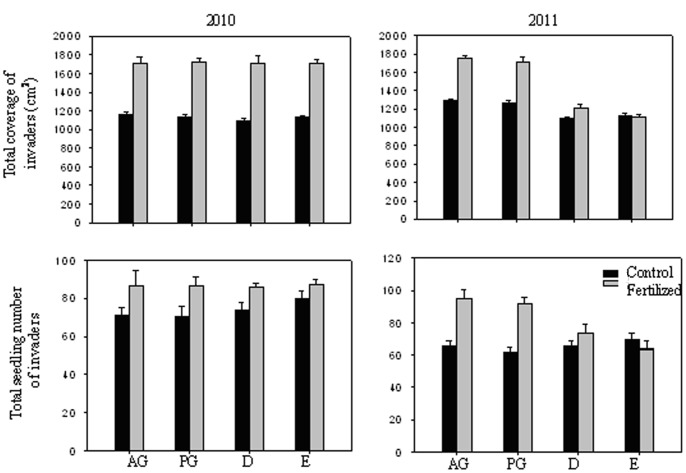
Effects of dominant functional group and fertilization on the coverage and seedling number of total invaders in 2010 and 2011. Dominant functional group treatments: AG – annual grass dominated pots. PG – perennial grass dominated pots, D – deciduous shrub or arbor dominated pots and E – evergreen shrub or arbor dominated pots.

Experimental fertilization significantly increased the total coverage and seedling number of invaders in 2010, with these increases persisted in 2011 (Table 3, Figure 1). However, for pots dominated by D and E, fertilization did not significantly increase the total coverage and seedling number of invaders in 2011. Dominant functional group had no significant effect on the total coverage and seedling number of invaders in 2010. However, invaders had more coverage and seedling numbers in pots dominated by AG and PG than those dominated by D and E in 2011. Moreover, the interaction of fertilization and dominant functional group also had a significant effect on total coverage and seedling number of invaders in 2011.

**Table 3:** Results from two-way ANOVA of effects of dominant functional group (D) and fertilization (F) treatments on the coverage and seedling number of invaders in pots in 2010 and 2011.

	Coverage			Number of seedlings		
	MS	F	P	MS	F	P
2010						
D	2222.19	0.90	0.45	59.47	2.74	0.06
F	4104535.79	1653.61	<0.001	1875.00	86.34	<0.001
D×F	2121.62	0.85	0.47	47.06	2.17	0.11
Error	2482.17			21.72		
2011						
D	545145.42	492.48	<0.001	481.19	25.96	<0.001
F	745751.04	673.71	<0.001	2806.02	151.37	<0.001
D×F	174719.39	157.84	<0.001	944.80	50.97	<0.001
Error	1106.94			18.54		

**Figure 2:**
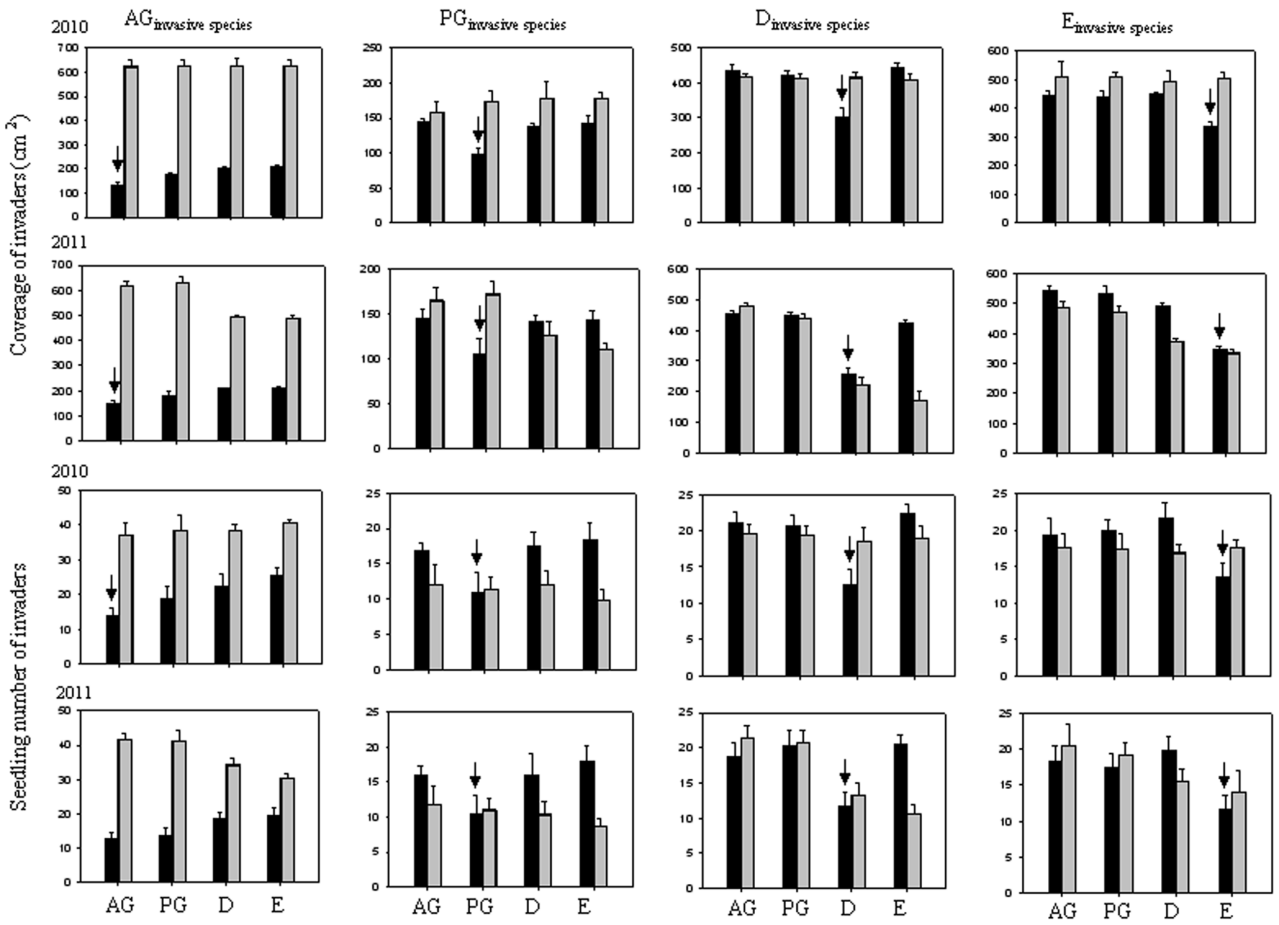
The coverage and seedling number of different functional groups of invaders in the pots dominated by different dominant functional groups in 2010 and 2011. Dominant functional group treatments: AG – annual grass dominated pots, PG – perennial grass dominated pots, D – deciduous shrub or arbor dominated pots and E – evergreen shrub or arbor dominated pots. AG_invasive species_ – the invader belong to annual grass. PG_invasive species_ – the invader belong to perennial grass. D_invasive species_ – the invader belong to deciduous shrub or arbor. E_invasive species_ – the invader belong to evergreen shrub or arbor. The legends are as given in Figure 1. Arrows (↓) indicate that the coverage and seedling number of invaders had lower values in the pots dominated by same functional group than those dominated by other functional group.

When invaders were grouped into functional groups there was a significant association between invaders and dominant functional group in control pots (Figure 2). Consistent with the hypothesis of limiting similarity, invaders had the lowest coverage and seedling number in control pots dominated by the same functional group. However, the limiting similarity disappeared in fertilized pots. Moreover, all four functional groups of invaders had lower coverage and seedling numbers in pots dominated by D and E than those dominated by AG and PG in 2011.

**Figure 3:**
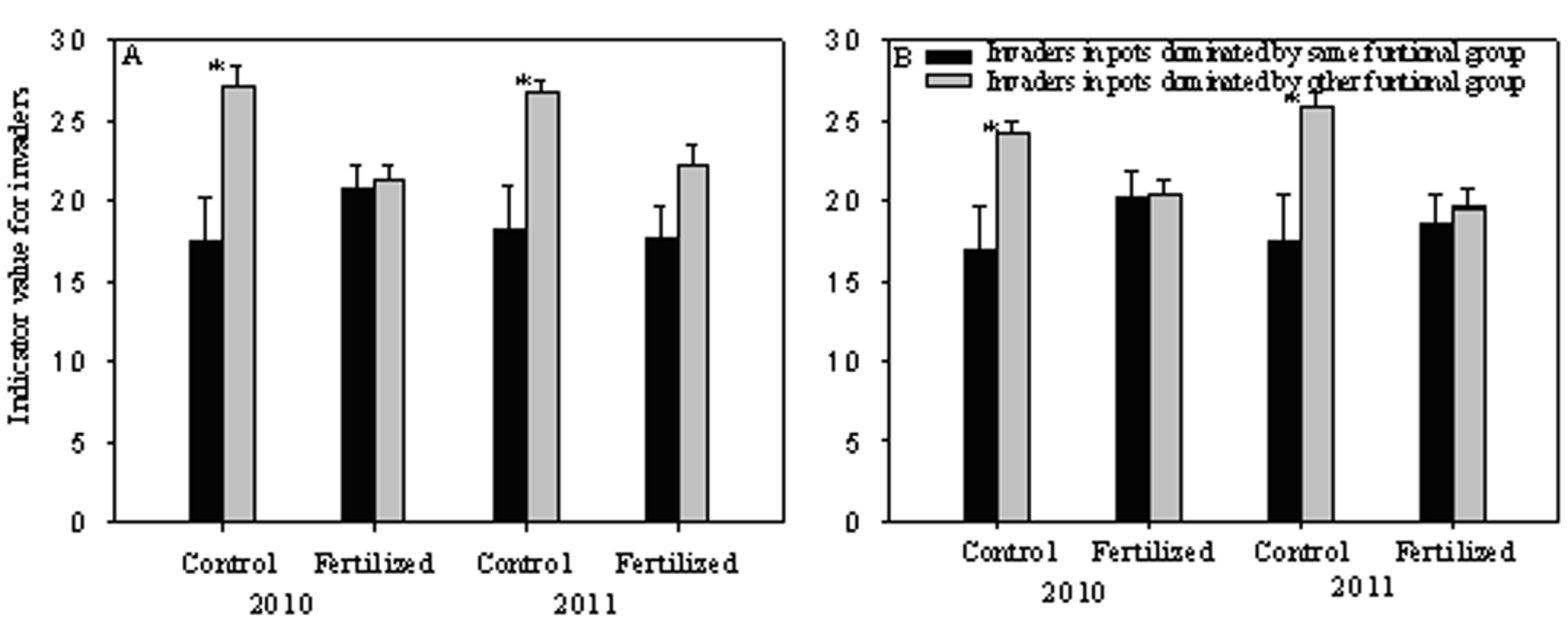
The indicator value (IV) of invaders for coverage (A) and seedling number (B) in the pots dominated by same functional group and the pots dominated by other functional group in 2010 and 2011. Higher indicator values represent higher colonization success. *indicate significant difference between the pots dominated by same functional group and the pots dominated by other functional group.

Although limiting similarity applied at functional group level in control pots, individual invaders of the same functional group did not always match this pattern in control pots (Table 4). For example, CG had the highest coverage in control pots dominated by PG but did not have the lowest coverage in control pots dominated by the same functional group. However, compared with fertilized pots, there were still more individual invaders with the least coverage and seedling numbers in control pots dominated by the same functional group. In general, invaders had lower indicator values in control pots dominated by the same than by another functional group (Figure 3, coverage: t_2010_ = –3.369, df = 78, P = 0.001; t_2011_ = –4.234, df = 78, P<0.001; seedling number: t_2010_ = –4.918, df = 78, P<0.001; t_2011_ = –4.259, df = 78, P<0.001). Similar to the functional group level, most individual invader species had the highest coverage in fertilized pots dominated by AG or PG in 2011, while nearly all species showed no differences in seedling numbers among pots dominated by a different functional group.

**Table 4:** Results from the indicator species analysis for the control and fertilized pots of 2010 and 2011.

	Coverage				Number of seedling			
	2010		2011		2010		2011	
	Control	Fertilized	Control	Fertilized	Control	Fertilized	Control	Fertilized
CA	D(34.5**)↓	ns	D(30.3*)↓	PG(29.9**)	ns↓	ns	E(36.1*)↓	PG(29.3*)
CT	E(31.8*)↓	ns	ns↓	AG(27.6*)	ns↓	ns	ns↓	ns
CG	PG(27.2*)	ns	PG(26.9*)	AG(27.1*)	PG(31.4*)	ns	ns	ns
BB	E(90.3**)↓	ns	D(34.4**)	PG(28.1**)	D(37.9**)↓	ns	ns	ns
TA	AG(28.4**)	ns	AG(28.7**)	AG(30.8**)	ns	ns	ns	ns
TAM	PG(50.5**)	ns	PG(47.6**)	ns	PG(54.1**)	ns	PG(54.8**)	ns
OC	AG(27.7*)↓	ns	PG(27.6*)	ns	ns↓	ns	ns	ns
RJ	AG(31.5*)↓	ns	ns↓	ns	ns↓	ns	ns↓	ns
PP	E(31**)↓	ns	AG(30.6*)↓	ns	ns↓	ns	ns↓	ns
GT	D(34.2*)	E(26.4**)	D(33.7**)↓	AG(32**)	D(35.9*)	ns	D(36.8*)↓	AG(31.5**)
RC	E(32.6*)↓	ns	AG(33.8**)↓	AG(32.2**)	AG(34.6*)↓	ns	ns↓	ns
VN	D(26.8*)	ns	PG(27*)	PG(33.8*)	ns	ns	ns	AG(32.8*)
LC	E(29.6*)↓	AG(26.4**)	PG(31.4**)↓	AG(38.1**)	ns↓	ns	ns↓	ns
HM	D(27.6**)	ns	ns	AG(47.9**)	ns	ns	ns	AG(42.9**)
EJ	AG(37.7**)↓	ns	AG(36.3**)↓	AG(40.3**)	ns↓	ns	ns↓	AG(39.6*)
PT	ns↓	ns	D(29.1*)↓	AG(28.2**)↓	ns↓	ns	D(31.6*)↓	ns
SB	D(28.8*)↓	ns	AG(30.3**)↓	ns	ns↓	ns	ns↓	ns
PS	ns↓	ns	ns↓	AG(28.1*)↓	D(31.3*)↓	ns	ns↓	ns
EO	ns	ns	ns	ns	ns	ns	ns	ns
ND	ns	AG(32.9**)	ns	AG(50.2**)	ns	ns	ns	ns

Significant relationships between the coverage and seedling number of each invader and certain dominant functional group treatment are shown for having the highest coverage and seedling number in the treatment. Numbers in parentheses are the indicator values (*IV*). Significance values are calculated based on 1000 randomizations in a Monte Carlo simulation, with *P<0.05, **P<0.01 and ns not significant (P>0.05). Species abbreviations are as given in Table 2. Arrows (↓) indicate that the coverage and seedling number of invaders had lower values in the pots dominated by the same than by another functional group, which indicates limiting similarity.

**Figure 4:**
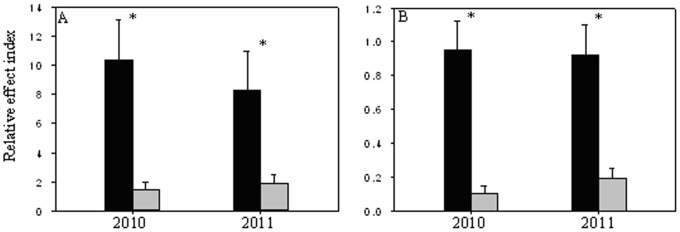
The relative effect index (REI) IV of invaders for coverage (A) and seedling number (B) in the pots dominated by same functional group and the pots dominated by other functional group in 2010 and 2011. Higher values of REI indicate more positive effect of fertilization on the colonization of invaders. *indicate significant difference between the pots dominated by same functional group and the pots dominated by other functional group. The legends are as given in Figure 3.

Two-way ANOVA showed that the effects of fertilization on coverage and seedling number exhibited great differences among individual invader species, and had a stronger influence than the dominant functional group (Tables S2 and S3 in File S1). However, invaders generally had higher REI in pots dominated by the same than by another functional group (Figure 4, coverage: t_2010_ = 4.729, df = 478, P<0.001; t_2011_ = 3.190, df = 478, P = 0.002; seedling number: t_2010_ = 6.601, df = 478, P<0.001; t_2011_ = 5.288, df = 478, P<0.001). ANCOVA showed that the REI for coverage of many individual invader species was also affected by light availability and other invaders of the same functional group (Table S4 in File S1), while they nearly had no significant effect on REI for seedling numbers of all invader species (Table S5 in File S1).

## Discussion

The results showed that fertilization significantly reduced the invasion resistance of experimental plant communities to total invaders. However, in contrast to expectations, the dominant functional group had little influence on the invasion resistance of total invaders in 2010. In 2011, mostly likely owing to lower light transmittance in pots dominated by D and E (*F*_3, 20_ = 137.64, P<0.001, data not shown), they had higher invasion resistance than pots dominated by AG and PG. Prior studies have shown that abiotic factors (e.g. light, nitrogen availability and soil moisture) regulated invasion success at the seedling establishment stage [38]–[40]. Consequently, it is most likely that dominant functional group should directly affect the resource availability, thereby indirectly influencing establishment of invader seedlings. We speculate that the lack of a significant effect of dominant functional group on invasion resistance to total invaders in 2010 may be caused by little effect of dominant functional group on the total amounts of soil nitrogen and light availability during the short establishment time.

Consistent with expectation, dominant functional group, independent of functional group identity, significantly affected the composition of invaders in control pots. However, invaders exhibited different invasion patterns: some invaders had less success in pots dominated by the same functional group; however, others did not, and even had more success in pots dominated by the same functional group. These different invasion patterns may be attributed to species differences within functional groups [8], [41], interactions among invaders [14] and other coexisting mechanisms, such as facilitation [42]. Although many factors influence the limiting similarity, when grouped into functional groups, invaders in pots dominated by the same functional group generally had less success than those in pots dominated by another functional group. In the present study, adding seeds of many invaders may have created seedling competition effects, but they also give a more complete picture of how a community may respond to invaders [8], [27], [38]. Our results indicate that limiting similarity should be more applicable at functional group level when predicting community succession and invasion resistance.

Although fertilization reduced the invasion resistance of communities to total invaders, its effects exhibited great differences for individual invaders. The results of REI for coverage showed that the effect of fertilization was mainly influenced by dominant functional group, light availability and other invaders of the same functional group. It is most likely that the influence of light availability and other invaders of the same functional group led to no significant associations between the success of invaders and dominant functional group in fertilized pots. However, fertilization generally led to more success of invaders in pots dominated by the same than by another functional group. We speculate that the invasion potential of invaders was more restrained in pots dominated by the same functional group, and competition release by fertilization [29] would lead to more invasion success for these invaders. Because the interaction of invaders was not controlled, the mechanism of the effect of invaders of the same functional group was not directly determined. However, the effect of fertilization on seedling numbers of invader species seems independent of the influence of light availability and other invader species.

The results showed that dominant functional group had no significant effect on light availability in 2010 but had a significant effect in 2011, which significantly influenced the invasion resistance of communities. Consequently, the effect of dominant functional group on invasion resistance most likely depends on time, especially for early successional communities. In the present study, the amounts of soil nitrogen were controlled. However, in natural communities, the temporal changes of soil nitrogen with the dominant functional group may also influence invasion patterns. More importantly, the coverage of invaders was more sensitive to temporal changes in resource availability than seedling numbers of invaders. Consequently, the temporal change of invader traits (from seedling to adult) [1], [11], [14], the temporal change of the effect of dominant functional group on resource availability and the responses of invader traits to changes in resource availability may have interaction effects on the invasion resistance of dominant functional groups.

Studies of invasion usually concern non-native invaders colonizing communities with which they have had no previous interactions [27]; however, native species were used as invaders in the present study. Despite somewhat different focuses, the ecological principles (e.g. niche limitation and competition for resources) underlying both types of studies are the same [27]. Thus, experiments using either native or non-native species as invader species are both pertinent to explaining which traits characterize the invasibility of communities. Although we only examined two years of establishment of invader species, seedling establishment is a key life-stage for invaders [43]–[45]. Understanding the effect of initial environmental ‘filters’, the resident dominant functional group and resource availability on invasion may aid in the control of natural or human-induced invasions.

## Supporting Information

File S1 **Contains: Table S1** The spontaneously colonized species in experimental pots. **Table S2** Results of two-way ANOVA for the effects of fertilization and dominant functional group on the coverage of each invader. Significant variables (*P*<0.05) are in bold. Arrows indicate significant increase (↑) or decrease (↓) of coverage of invader with fertilization. The values out of and in the bracket is the results of 2010 and 2011 respectively. **Table S3** Results of two-way ANOVA for the effects of fertilization and dominant functional group on the seedling number of each invader. Significant variables (*P*<0.05) are in bold. Arrows indicate significant increase (↑) or decrease (↓) of seedling number of invader with fertilization. The values out of and in the bracket is the results of 2010 and 2011 respectively. **Table S4** Results for ANCOVA of effects of dominant functional group on the relative effect index (REI) of invader coverage with the change of light availability, the coverage change of invaders of same functional group and the coverage change of invaders of other functional group as covariate. Significant variables (*P*<0.05) are in bold. The values out of and in the bracket is the results of 2010 and 2011 respectively. **Table S5** Results for ANCOVA of effects of dominant functional group on the relative effect index (REI) of invader seedling number with the change of light availability, the seedling number change of invaders of same functional group and the seedling number change of invaders of other functional group as covariate. Significant variables (*P*<0.05) are in bold. The values out of and in the bracket is the results of 2010 and 2011 respectively. **Figure S1** The changes of light transmittance percentage in different dominant functional group treatments. Dominant functional group treatments: A) annual grass dominated pots. B) perennial grass dominated pots, C) deciduous shrub or arbor dominated pots and D) evergreen shrub or arbor dominated pots. The average value of six replicated pots was used. Light transmittance percentage was measured every month at midday on cloud free days, and was measured 20 times in total (2009-Aug to 2010-May and 2010-Aug to 2011-May).
(DOC)
