# Supplementary material for: Dominant Functional Group Effects on the Invasion Resistance at Different Resource Levels
Source: PLoS One. 2013 Oct 22;8(10):e77220. doi: 10.1371/journal.pone.0077220 (PMC3805585; doi:10.1371/journal.pone.0077220)
Supplement: File S1 — Contains: Table S1 The spontaneously colonized species in experimental pots. Table S2 Results of two-way ANOVA for the effects of fertilization and dominant functional group on the coverage of each invader. Significant variables (P<0.05) are in bold. Arrows indicate significant increase (↑) or decrease (↓) of coverage of invader with fertilization. The values out of and in the bracket is the results of 2010 and 2011 respectively. Table S3 Results of two-way ANOVA for the effects of fertilization and dominant functional group on the seedling number of each invader. Significant variables (P<0.05) are in bold. Arrows indicate significant increase (↑) or decrease (↓) of seedling number of invader with fertilization. The values out of and in the bracket is the results of 2010 and 2011 respectively. Table S4 Results for ANCOVA of effects of dominant functional group on the relative effect index (REI) of invader coverage with the change of light availability, the coverage change of invaders of same functional group and the coverage change of invaders of other functional group as covariate. Significant variables (P<0.05) are in bold. The values out of and in the bracket is the results of 2010 and 2011 respectively. Table S5 Results for ANCOVA of effects of dominant functional group on the relative effect index (REI) of invader seedling number with the change of light availability, the seedling number change of invaders of same functional group and the seedling number change of invaders of other functional group as covariate. Significant variables (P<0.05) are in bold. The values out of and in the bracket is the results of 2010 and 2011 respectively. Figure S1 The changes of light transmittance percentage in different dominant functional group treatments. Dominant functional group treatments: A) annual grass dominated pots. B) perennial grass dominated pots, C) deciduous shrub or arbor dominated pots and D) evergreen shrub or arbor dominated pots. The average value of six re [file pone.0077220.s001.doc]

**Table S1** The spontaneously colonized species in experimental pots.

|  | Invader species | Abbreviation | Family |
| --- | --- | --- | --- |
| Annual grass |  |  |  |
|  | *Youngia japonica* (Linn.) DC | YJ | Compositae |
|  | *Erigeron annuus* (Linn.) Pers | EA | Compositae |
|  | *Veronica didyma* Tenore | VD | Scrophulariaceae |
|  | *Mazus pumilus* (Burm.f.) Van Steenis | MP | Scrophulariaceae |
|  | *Amaranthus tricolor* Linn | AT | Amaranthaceae |
|  | *Digitaria ciliaris* (Retz.) Koel | DC | Papaveraceae |
| Perennial grass |  |  |  |
|  | *Macleaya cordata* (Willd.) R.Br | MC | Saxifragaceae |
|  | *Penthorum chinense* Pursh | PC | Gramineae |
| Leguminosae |  |  |  |
|  | *Crotalaria spectabilis* Roth. | CS | Leguminosae |
|  | *Lespedeza pilosa* (Thunb.) Sieb.et Zucc. | LP | Leguminosae |
|  | *Glycine soja* Sieb. et Zucc | GS | Leguminosae |
| Fujimoto |  |  |  |
|  | *Porana racemosa* Roxb | PR | Convolvulaceae |

**Table S2** Results of two-way ANOVA for the effects of fertilization and dominant functional group on the coverage of each invader. Significant variables (*P* < 0.05) are in bold. Arrows indicate significant increase (↑) or decrease (↓) of coverage of invader with fertilization. The values out of and in the bracket is the results of 2010 and 2011 respectively.

|  | Fertilization | | Dominant functional group | | Interaction | |
| --- | --- | --- | --- | --- | --- | --- |
|  | F | P | F | P | F | P |
| CA | 686.93↑(629.95↑) | **<0.001(<0.001)** | 7.66 (10.39) | **<0.001 (<0.001)** | 3.99 (27.79) | **0.014(<0.001)** |
| CT | 644.86↑(1355.40↑) | **<0.001(<0.001)** | 4.40 (11.31) | **0.009**(**<0.001**) | 9.68 (42.98) | **<0.001** (**<0.001**) |
| CG | 1801.61↑(2821.28↑) | **<0.001(<0.001)** | 1.03 (13.23) | 0.387(**<0.001**) | 1.85 (15.27) | 0.152(**<0.001**) |
| BB | 1924.95↑(2166.86↑) | **<0.001(<0.001)** | 12.20 (3.76) | **<0.001** (**0.018**) | 20.46 (54.35) | **<0.001** (**<0.001**) |
| TA | 2878.18↑(2759.68↑) | **<0.001(<0.001)** | 0.83 (117.76) | 0.484(**<0.001**) | 2.61 (62.60) | **0.001**(**<0.001**) |
| TAM | 15.84↓(23.36↓) | **<0.001** **(<0.001)** | 5.63 (11.33) | **0.002(<0.001)** | 6.24 (12.43) | **<0.001** (**<0.001**) |
| OC | 176.07↓(193.27↓) | **<0.001(<0.001)** | 1.00 (1.24) | 0.403(0.305) | 2.54 (2.03) | 0.069(0.125) |
| RJ | 77.19↓(89.09↓) | **<0.001**(**<0.001**) | 7.20 (6.84) | **<0.001 (0.001)** | 7.71 (8.53) | **<0.001** (**<0.001**) |
| PP | 161.69↓(145.36↓) | **<0.001(<0.001)** | 6.03 (6.12) | **0.002(0.002)** | 11.47 (11.48) | **<0.001** (**<0.001**) |
| GT | 6860.70↑(2806.47↑) | **<0.001(<0.001)** | 40.73 (63.24) | **<0.001**(**<0.001**) | 31.81 (153.75) | **<0.001** (**<0.001**) |
| RC | 33.51↓(198.71↓) | **<0.001 (<0.001)** | 71.16 (180.12) | **<0.001(<0.001)** | 47.21 (61.65) | **<0.001** (**<0.001**) |
| VN | 167.35↑(16.30↓) | **<0.001(<0.001)** | 7.17 (165.75) | **<0.001 (<0.001)** | 2.99 (117.02) | **0.042**(**<0.001**) |
| LC | 48.38↑(4.25↓) | **<0.001 (0.046)** | 41.56 (288.54) | **<0.001(<0.001)** | 42.25 (105.46) | **<0.001** (**<0.001**) |
| HM | 267.37↓(694.48↓) | **<0.001(<0.001)** | 3.81 (20.23) | **0.019(<0.001)** | 0.67 (33.48) | 0.571(**<0.001**) |
| EJ | 9.58↓ (0.17) | **0.004**(0.682) | 35.48 (41.06) | **<0.001 (<0.001)** | 54.80 (13.54) | **<0.001** (**<0.001**) |
| PT | 1005.73↑(753.37↑) | **<0.001(<0.001)** | 9.22 (96.54) | **<0.001 (<0.001)** | 20.15 (24.25) | **<0.001** (**<0.001**) |
| SB | 853.79↓(1934.84↓) | **<0.001(<0.001)** | 7.96 (91.42) | **<0.001 (<0.001)** | 8.44 (51.08) | **<0.001** (**<0.001**) |
| PS | 1378.65↑(1102.80↑) | **<0.001(<0.001)** | 9.13 (83.78) | **<0.001 (<0.001)** | 8.47 (6.11) | **<0.001** (**0.002**) |
| EO | 399.01↓(659.77↓) | **<0.001(<0.001)** | 0.69 (2.44) | 0.558(0.078) | 0.83 (0.29) | 0.482(0.826) |
| ND | 1010.89↓(1074.72↓) | **<0.001(<0.001)** | 0.06 (5.64) | 0.978(**0.003**) | 0.41 (8.23) | 0.721(**<0.001**) |

**Table S3** Results of two-way ANOVA for the effects of fertilization and dominant functional group on the seedling number of each invader. Significant variables (*P* < 0.05) are in bold. Arrows indicate significant increase (↑) or decrease (↓) of seedling number of invader with fertilization. The values out of and in the bracket is the results of 2010 and 2011 respectively.

|  | Fertilization |  | Dominant functional group | | Interaction |  |
| --- | --- | --- | --- | --- | --- | --- |
|  | F | P | F | P | F | P |
| CA | 359.25↑(278.11↑) | **<0.001(<0.001)** | 11.49 (2.50) | **<0.001(**0.073**)** | 0.43 (13.82) | 0.728**(<0.001)** |
| CT | 101.96↑(280.66↑) | **<0.001(<0.001)** | 11.27 (4.40) | **<0.001** (**0.009**) | 8.43 (24.15) | **<0.001** (**<0.001**) |
| CG | 77.19↑(311.35↑) | **<0.001(<0.001)** | 6.99 (0.90) | **0.001**(0.449) | 3.39 (4.50) | **0.027**(**0.008**) |
| BB | 85.74↑(285.12↑) | **<0.001(<0.001)** | 13.91 (7.46) | **<0.001** (**<0.001**) | 14.93 (19.66) | 0.875(**<0.001**) |
| TA | 111.72↑(130.79↑) | **<0.001(<0.001)** | 0.37 (3.87) | 0.775(**0.016**) | 0.22 (8.05) | **0.001**(**<0.001**) |
| TAM | 14.14↓(13.33↓) | **0.001**(**0.001**) | 4.23 (4.66) | **0.011(0.007)** | 7.15 (5.95) | 0.360(**0.002**) |
| OC | 304.46↓(234.23↓) | **<0.001(<0.001)** | 0.45 (0.83) | 0.717(0.485) | 1.10 (1.74) | **<0.001** (0.174) |
| RJ | 113.62↓(70.24↓) | **<0.001**(**<0.001**) | 10.09 (7.88) | **<0.001 (<0.001)** | 11.69 (9.34) | **0.014**(**<0.001**) |
| PP | 53.51↓(58.54↓) | **<0.001(<0.001)** | 3.82 (3.49) | **0.017(0.024)** | 4.01 (6.64) | **<0.001** (**0.001**) |
| GT | 161.64↑(162.56↑) | **<0.001(<0.001)** | 11.70 (11.37) | **<0.001** (**<0.001**) | 14.34 (25.02) | **<0.001** (**<0.001**) |
| RC | 0.02 (3.82) | 0.866(0.057) | 16.36 (18.26) | **<0.001 (<0.001)** | 20.37 (14.46) | **<0.001** (**<0.001**) |
| VN | 16.65↑(9.25↑) | **<0.001(0.004)** | 0.62 (9.94) | 0.602(**<0.001**) | 1.72 (20.74) | 0.177(**<0.001**) |
| LC | 3.33 (1.32) | 0.075(0.257) | 12.48 (12.35) | **<0.001(<0.001)** | 6.8 (6.08) | **0.001**(**0.002**) |
| HM | 27↓(36.56↓) | **<0.001**(**<0.001**) | 0.62 (5.72) | 0.600**(0.002)** | 1.22 (2.47) | 0.314(0.075) |
| EJ | 9.27↓(8.10↓) | **0.004**(0.007) | 6.81 (16.96) | **0.001(<0.001)** | 6.85 (4.81) | **0.001** (**0.006**) |
| PT | 101.76↑(121.42↑) | **<0.001(<0.001)** | 4.53 (14.13) | **0.008(<0.001)** | 9.52 (3.49) | **<0.001** (**0.024**) |
| SB | 160.14↓(177.96↓) | **<0.001(<0.001)** | 4.06 (6.79) | **0.013(0.001)** | 2.89 (6.48) | **0.047** (**0.001**) |
| PS | 151.15↑(165.08↑) | **<0.001(<0.001)** | 4.70 (13.63) | **0.007(<0.001)** | 7.16 (3.60) | **0.001** (**0.021**) |
| EO | 167.48↓(141.32↓) | **<0.001(<0.001)** | 0.17 (0) | 0.910(1.000) | 0.48 (0.53) | 0.698(0.658) |
| ND | 54.91↓(46.87↓) | **<0.001(<0.001)** | 1.07 (2.91) | 0.371(**0.046**) | 1.12 (1.45) | 0.347(0.240) |

**Table S4** Results for ANCOVA of effects of dominant functional group on the relative effect index (REI) of invader coverage with the change of light availability, the coverage change of invaders of same functional group and the coverage change of invaders of other functional group as covariate. Significant variables (*P* < 0.05) are in bold. The values out of and in the bracket is the results of 2010 and 2011 respectively.

|  | Dominant functional group | | Light | | Invaders of same funtional group | | Invaders of other functional group | |
| --- | --- | --- | --- | --- | --- | --- | --- | --- |
|  | F | P | F | P | F | P | F | P |
| CA | 1.727(0.708) | 0.199(0.561) | 0.433(0.158) | 0.518(0.696) | 0.408(0.024) | 0.531(0.879) | 0.001(0.012) | 0.970(0.915) |
| CT | 0.492(3.330) | 0.692**(0.044)** | 0.345(0.061) | 0.564(0.808) | 2.919(6.059) | 0.105(**0.025**) | 0.006(5.086) | 0.934**(0.038)** |
| CG | 6.500(8.050) | **0.003(0.001)** | 0.001(0.166) | 0.989(0.689) | 2.773(0.698) | 0.114(0.415) | 0.141(0.047) | 0.711(0.831) |
| BB | 3.442(8.601) | **0.047**(**0.001**) | 0.001(3.694) | 0.975**(0.048)** | 0.098(1.985) | 0.757(0.177) | 0.561(0.784) | 0.463(0.388) |
| EA | 12.034(1.914) | **<0.001**(0.166) | 0.101(0.004) | 0.754(0.953) | 0.395(2.531) | 0.537(0.130) | 3.485(0.116) | 0.079(0.738) |
| TAM | 3.614(3.349) | **0.034**(**0.041**) | 0.021(0.714) | 0.884(0.410) | 12.666(5.038) | **0.002(0.038)** | 0.006(0.002) | 0.935(0.968) |
| OC | 3.315(0.607) | **0.039**(0.620) | 0.001(0.937) | 0.997(0.347) | 9.419(1.983) | **0.006**(0.177) | 2.352(0.105) | 0.143(0.750) |
| RJ | 0.029(0.860) | 0.993(0.481) | 4.408(5.930) | **0.042(0.026)** | 1.522(4.922) | 0.234(**0.041**) | 1.575(3.640) | 0.226(0.073) |
| MC | 0.377(0.608) | 0.770(0.619) | 0.166(0.248) | 0.688(0.625) | 0.4776(0.109) | 0.498(0.745) | 0.340(0.035) | 0.567(0.854) |
| PC | 2.546(0.993) | **0.090**(0.420) | 4.303(0.009) | **0.046**(0.927) | 0.175(0.765) | 0.680(0.394) | 0.086(0.356) | 0.771(0.559) |
| RC | 3.205(3.356) | **0.049**(**0.042**) | 0.533(0.482) | 0.475(0.497) | 2.300(4.710) | 0.147(**0.044**) | 0.722(1.707) | 0.407(0.209) |
| VN | 4.148(3.472) | **0.022(0.039)** | 0.002(1.218) | 0.965(0.285) | 5.749(6.189) | **0.028(0.024)** | 0.539(2.693) | 0.472(0.119) |
| LC | 9.209(3.947) | **<0.001(0.026)** | 0.626(0.039) | 0.439(0.845) | 13.497(9.194) | **0.001(0.008)** | 2.565(0.256) | 0.127(0.620) |
| HM | 1.858(5.018) | 0.174**(0.011)** | 0.067(4.090) | 0.798(**0.035**) | 4.746(1.488) | **0.043**(0.239) | 3.464(0.385) | 0.080(0.543) |
| EJ | 3.239(8.000) | **0.048(0.002)** | 4.422(0.001) | **0.040**(0.994) | 13.364(29.925) | **0.001(<0.001)** | 0.278(5.221) | 0.604(**0.035**) |
| PT | 9.713(5.618) | **<0.001(0.007)** | 0.008(3.757) | 0.926(**0.042**) | 7.483(4.840) | **0.014(0.042)** | 0.693(1.443) | 0.416(0.246) |
| SB | 1.479(5.453) | 0.255(**0.008**) | 4.107(0.833) | **0.050**(0.374) | 2.220(16.732) | 0.154**(0.001)** | 0.670(2.418) | 0.424(0.138) |
| PS | 8.230(1.495) | **0.001**(0.252) | 4.221(3.712) | **0.049**(**0.046**) | 7.778(0.907) | **0.012**(0.354) | 0.460(0.845) | 0.506(0.371) |
| EO | 1.661(0.135) | 0.212(0.938) | 0.055(0.583) | 0.817(0.455) | 7.515(0.001) | **0.139**(0.974) | 0.037(0.002) | 0.847(0.969) |
| ND | 5.499(1.616) | **0.007**(0.225) | 0.362(0.647) | 0.554(0.432) | 0.404(2.743) | 0.533(0.116) | 2.796(0.840) | 0.112(0.372) |

**Table S5** Results for ANCOVA of effects of dominant functional group on the relative effect index (REI) of invader seedling number with the change of light availability, the seedling number change of invaders of same functional group and the seedling number change of invaders of other functional group as covariate. Significant variables (*P* < 0.05) are in bold. The values out of and in the bracket is the results of 2010 and 2011 respectively.

|  | Dominant functional group | | Light | | Invaders of same funtional group | | Invaders of other functional group | |
| --- | --- | --- | --- | --- | --- | --- | --- | --- |
|  | F | P | F | P | F | P | F | P |
| CA | 0.923(2.825) | 0.451(0.070) | 0.002(1.293) | 0.964(0.271) | 0.420(0.615) | 0.5526(0.444) | 0.244(0.278) | 0.627(0.605) |
| CT | 11.594(10.707) | **<0.001(<0.001)** | 0.925(0.023) | 0.350(0.882) | 0.003(0.008) | 0.958(0.929) | 2.512(0.105) | 0.131(0.749) |
| CG | 5.461(0.998) | **0.008**(0.418) | 8.615(0.011) | **0.009**(0.918) | 0.141(0.490) | 0.712(0.493) | 0.778(1.438) | 0.390(0.247) |
| BB | 8.189(14.496) | **0.001(<0.001)** | 0444(2.361) | 0.514(0.143) | 3.984(1.743) | 0.0.062(0.204) | 0.566(0.216) | 0.462(0.648) |
| EA | 1.777(1.259) | 0.190(0.320) | 0.346(0.002) | 0.564(0.965) | 5.376(0.008) | **0.033**(0.929) | 0.117(1.486) | 0.737(0.240) |
| TAM | 0.597(1.681) | 0.626(0.209) | 0.221(0.519) | 0.645(0.481) | 0.111(0.075) | 0.743(0.787) | 2.053(5.465) | 0.170**(0.032)** |
| OC | 0.216(0.598) | 0.884(0.625) | 0.304(1.914) | 0.558(0.184) | 0.190(0.001) | 0.558(0.982) | 0.981(0.114) | 0.336(0.740) |
| RJ | 2.788(3.437) | 0.072**(0.041)** | 3.391(7.171) | 0.083**(0.016)** | 0.678(0.172) | 0.422(0.684) | 0.099(0.012) | 0.757(0.912) |
| MC | 1.541(4.072) | 0.240**(0.024)** | 0.629(1.631) | 0.439(0.219) | 0.023(0.017) | 0.881(0.898) | 0.516(0.590) | 0.882(0.453) |
| PC | 24.678(12.096) | **<0.001(<0.001)** | 2.205(0.178) | 0.056(0.678) | 0.252(2.086) | 0.622(0.167) | 1.866(0.145) | 0.190(0.709) |
| RC | 9.614(9.426) | **0.001**(**0.001**) | 0.070(0.049) | 0.795(0.827) | 2.390(2.973) | 0.140(0.103) | 3.762(2.191) | 0.069(0.157) |
| VN | 1.706(2.445) | 0.204(0.099) | 0.218(0.471) | 0.647(0.502) | 1.163(0.911) | 0.296(0.353) | 1.952(0.755) | 0.180(0.397) |
| LC | 4.775(3.227) | **0.014(0.049)** | 1.186(0.007) | 0.291(0.936) | 1.866(0.740) | 0.190(0.402) | 2.004(0.037) | 0.175(0.850) |
| HM | 0.670(0.334) | 0.582(0.801) | 0.650(1.615) | 0.431(0.221) | 0.702(0.539) | 0.414(0.473) | 2.193(0.086) | 0.157(0.773) |
| EJ | 3.076(0.910) | 0.056(0.457) | 0.033(1.016) | 0.858(0.328) | 1.136(0.263) | 0.301(0.615) | 0.053(0.537) | 0.820(0.474) |
| PT | 17.769(0.663) | **<0.001**(0.586) | 0.049(0.004) | 0.827(0.953) | 0.043(4.394) | 0.839(0.051) | 1.831(0.082) | 0.194(0.778) |
| SB | 0.671(0.628) | 0.581(0.607) | 1.870(0.037) | 0.189(0.850) | 0.484(3.199) | 0.496(0.082) | 0.530(0.476) | 0.447(0.479) |
| PS | 2.374(2.094) | 0.106(0.139) | 1.444(0.081) | 0.426(0.779) | 1.706(5.379) | 0.209(**0.033**) | 2.793(0.695) | 0.113(0.426) |
| EO | 1.224(0.495) | 0.331(0.690) | 1.451(1.105) | 0.245(0.308) | 0.280(0.001) | 0.604(0.995) | 3.608(0.365) | 0.075(0.554) |
| ND | 1.122(0.145) | 0.368(0.931) | 3.020(0.223) | 0.100(0.643) | 0.140(1.270) | 0.713(0.275) | 0.356(0.056) | 0.559(0.816) |

**Figure S1** The changes of light transmittance percentage in different dominant functional group treatments. Dominant functional group treatments: A) annual grass dominated pots, B) perennial grass dominated pots, C) deciduous shrub or arbor dominated pots and D) evergreen shrub or arbor dominated pots. The average value of six replicated pots was used. Light transmittance percentage was measured every month at midday on cloud free days, and was measured 20 times in total (Aug 2009 to May 2010 and Aug 2010 to May 2011).
